# Effects of Fermented *Caragana korshinskii* on Growth Performance, Antioxidant Status, Meat Quality, and Rumen Microbiota in Mongolian Sheep

**DOI:** 10.3390/ani16101473

**Published:** 2026-05-11

**Authors:** Guoqing Guo, Feng Tian, Yuan Gao, Jiuyue Li, Jianyong Liang, Yong Wang, Yang Liu, Shuyuan Xue

**Affiliations:** Inner Mongolia Academy of Agricultural Animal Husbandry Sciences, Hohhot 010018, China; 13404802050@163.com (G.G.); may5june6@yeah.net (Y.G.); lijy009@163.com (J.L.);

**Keywords:** fermented caragana, growth performance, Mongolian sheep, meat quality, rumen microbiota

## Abstract

*Caragana korshinskii* is an underutilized non-grain feed resource. This study investigated the optimal dietary inclusion level of fermented *Caragana korshinskii* for Mongolian lambs by assessing growth performance, antioxidant status, meat quality, fatty acid and amino acid profiles, and rumen microbiota. Thirty-six lambs were fed diets containing 0%, 10%, 15%, or 20% fermented *Caragana korshinskii* replacing *Leymus chinensis* hay for 105 days after a 10 d adaptation period. The 10% group exhibited higher final body weight, average daily gain, feed intake, and carcass weight (*p* < 0.05). All supplemented groups showed elevated serum T-AOC and reduced MDA (*p* < 0.05); furthermore, 10% supplementation increased CAT, and 10% and 15% supplementation increased SOD (*p* < 0.05). Meat quality improved in the 10% and 15% groups, with lower water loss, shear force, drip loss, L*, and b* (*p* < 0.05; *p* < 0.01), and higher a* (*p* < 0.05). Muscle nutrients remained unchanged. Amino acid alterations included increased Glu (all groups), Thr, Ile (15%, 20%), Leu (20%), and variable changes in Asp. Fatty acid analysis revealed higher C18:0, PUFA, C18:2n6c, C18:3n3 in 10% (*p* < 0.05; *p* < 0.01), but lower PUFA and C18:3n3 in the 15% and 20% groups (*p* < 0.05). Rumen 16S sequencing revealed higher abundances of *Thermodesulfobacteriota*, *Methanobacteriota*, *Methanobrevibacter* (10%, 20%), *Xylanibacter*, *Succiniclasticum* (15%, 20%), and lower *Rikenellaceae_RC9_gut_group* (20%) (*p* < 0.05; *p* < 0.01). Correlations linked rumen microbes to meat-related fatty acids and amino acids. In conclusion, supplementation with 10% fermented *Caragana korshinskii* optimally enhanced lamb growth, health, and meat quality through modulation of the rumen microbiota.

## Simple Summary

*Caragana korshinskii* is a shrub widely distributed in the arid and semi-arid regions of China. Although it is rich in protein, its high fiber content limits its digestibility when fed directly to livestock. In this study, the shrub was processed through microbial fermentation and evaluated at different dietary inclusion levels in the diets of Mongolian sheep. Results showed that adding 10% fermented *Caragana korshinskii* improved body weight gain, feed intake, antioxidant capacity, and meat tenderness and color. Rumen microbial analysis revealed that the fermented feed altered the rumen microbial community, and these changes were closely associated with the amino acid and fatty acid profiles in the muscle. Collectively, these findings indicate that including 10% fermented *Caragana korshinskii* in the diet can effectively enhance growth performance and meat quality in Mongolian sheep, supporting its utilization as a sustainable feed resource.

## 1. Introduction

The rapid development of the livestock sector has driven continuous expansion in both production scale and herd size, exacerbating the shortage of forage supply in recent years. This imbalance, commonly referred to as the Forage–Livestock Conflict (FLC), represents a critical manifestation of resource constraints. Between 2005 and 2015, China’s grazing sector generated an average of 82 million tonnes of FLC annually. Notably, 85–93% of this FLC was driven by domestic final demand. These findings indicate that the intensifying imbalance between forage supply and demand is not merely a production-side issue but rather a consumption-driven resource challenge, which has emerged as a major bottleneck constraining the sustainable development of grassland-based animal production [1]. This increasing forage supply and demand imbalance has exacerbated fodder scarcity. Against this backdrop, developing alternative forage resources has become a critical strategy to mitigate this shortfall. *Caragana korshinskii*, a perennial leguminous shrub widely distributed across the desert and semi-desert grasslands of northwestern China, represents one such promising resource. Owing to its strong adaptability, drought resistance, and cold tolerance, it has established itself as a valuable regional browse species for livestock [2]. *Caragana korshinskii* is abundant in protein and essential amino acids, and also contains a range of bioactive compounds, including alkaloids, flavonoids, and coumarins, making it a valuable roughage source for livestock [3]. However, its stems possess high levels of indigestible cellulose and lignin. Notably, with advancing maturity, lignification intensifies and crude fiber content further increases.

To enhance the utilization efficiency of *Caragana korshinskii*, microbial fermentation technology has been employed [4]. This process effectively degrades cellulose and lignin, thereby improving palatability and digestibility and increasing its feeding value. Fermented feed [5] refers to a functional feed produced by inoculating raw materials with specific microorganisms and composite enzymes, which converts them into nutrient-rich product containing microbial proteins, bioactive peptides, and amino acids. Such feed not only improves nutritional profiles but also promotes animal growth, maintains gut microbiota balance, and enhances immune function, offering considerable practical benefits in animal production.

This study aimed to systematically investigate the utilization of fermented *Caragana korshinskii* as a feed resource by examining the effects of different dietary inclusion levels on the growth performance and slaughter characteristics of Mongolian sheep. Beyond facilitating efficient conversion of Caragana resources and optimizing production costs, this study also investigated their impact on serum antioxidant profiles and the mechanisms underlying meat quality improvement. These findings are expected to provide a scientific basis for the large-scale utilization of *Caragana korshinskii* as a feed resource.

## 2. Materials and Methods

### 2.1. Preparation of Caragana korshinskii Haylage

The composition of *Leymus chinensis* hay and fermented *Caragana korshinskii* used in this study is shown in Table 1. The fermented *Caragana korshinskii* was obtained from Jiyuan Company (Siziwang Banner, Inner Mongolia, China). *Caragana korshinskii* was harvested at the late vegetative stage (early October), defined as the point when approximately 50% of leaves had turned yellow and begun to senesce, prior to the onset of full dormancy. The harvested material was harvested using a shrub harvester. The harvested plant materials were wilted in the field to about 95% DM, chopped into 8–10 cm pieces using a 4GM-200A self-propelled shrub combine harvester (Chinese Academy of Agricultural Mechanization Sciences, Beijing, China)., and subsequently inoculated with an in-house developed mixed inoculant consisting of lactic acid bacteria (*Lactobacillus plantarum*, CGMCC 1.2437) and *Bacillus subtilis* (CGMCC 1.15792). Inoculation was carried out by spraying the inoculants using a knapsack sprayer at a rate of ≥10^5^ CFU/g DM per strain (each strain applied individually, not as a mixture). The material was then thoroughly mixed and subsequently packed into plastic bags (1.5–3.6 m; Zibo Salite Packaging Products Co., Ltd., Shandong, China) at a density of 650 kg/m^3^. The bags were sealed and stored at 25 °C for 30 days prior to feeding.

### 2.2. Animals, Treatments and Feeding Experiment

A total of 36 5-month-old female Mongolian lambs with similar body weight (20.26 ± 1.90 kg) were randomly allocated into 12 pens measuring 3.0 m^2^, with three lambs per pen, providing a floor space allowance of 1.0 m^2^ per lamb. This stocking density falls within the recommended range for fattening lambs according to Chinese feeding standards (NY/T 816-2021, typically 0.5–1.0 m^2^ per animal). All animal procedures were approved by the Institutional Animal Care and Use Committee of the Inner Mongolia Academy of Agricultural and Animal Husbandry Sciences (protocol code: 2026-0018). The 12 pens were then randomly divided into 4 groups (3 pens per group). The four groups of lambs were randomly assigned to the Control (C) group, in which the lambs were fed a *Leymus chinensis* hay basal diet, or to one of the three treatment groups, in which the *Leymus chinensis* hay in the basal diet was replaced by the fermented *Caragana korshinskii* at 10% (CK10), 15% (CK15) or 20% (CK20). The experimental diets were formulated to meet the Nutrient Requirements of Meat Sheep (NY/T 816-2021) and consisted (DM basis) of 60% concentrate and 40% respective roughages. The concentrate was obtained from Inner Mongolia Mengtai Dadi Biotechnology Co., Ltd. (Hohhot, China). The diets (Table 2) were prepared as total mixed rations (TMR) at the time of feeding.

The feeding trial was conducted from August to November 2023, spanning a total of 115 d, including a 10-day adaptation period and a 105-day data collection phase. The trial took place at a breeding facility operated by Sino Company, located in Siziwang Banner. Lambs were pen-fed twice daily for ad libitum intake and had free access to water throughout the study. Orts were collected daily to determine average daily DM intake (ADMI). Lambs were weighed at 7:00 h after 16 h of fasting on the first and last day of the experimental period to determine live weight (LW) gain. Average daily gain (ADG) was calculated by dividing the LW with number of the days of the experiment. Feed conversion rate was calculated as ADMI/ADG.

### 2.3. Blood and Rumen Sampling and Determining Carcass Traits

Blood samples were aseptically collected from the jugular veins of all 36 lambs (9 lambs per group) on day 0 and day 105 of the experiment. Blood samples were collected into plain vacuum tubes without anticoagulant (Jiangsu Kangjian Medical Apparatus Co., Ltd., Taizhou, China) and allowed to clot at room temperature for 30 min. The samples were then centrifuged at 3000× *g* for 10 min, and the resulting serum was transferred into separate cryogenic vials and stored at −20 °C until analysis.

On the final morning of the experiment, one lamb was randomly selected from each pen (3 lambs per group, totaling 12 lambs) for rumen fluid sampling using a specialized rumen fluid collector inserted orally into the rumen [6]. The initial portion of rumen fluid was discarded, and approximately 20 mL was then transferred into separate cryovials, frozen in liquid nitrogen, and stored at −80 °C.

Upon completion of the feeding trial, 12 sheep (one from each pen) were randomly selected for slaughter. Slaughter was conducted at the Siziwang Banner Experimental Base of the Inner Mongolia Academy of Agricultural and Animal Husbandry Sciences, approximately 15 km from the feeding facility (transport time approximately 30 min). Lambs were fasted for 24 h with water withheld for the final 2 h before transport, and then exsanguinated by severing the jugular vein. All animal procedures in this study (including feeding, housing, blood sampling, rumen fluid sampling, and slaughter) were approved by the Institutional Animal Care and Use Committee of the Inner Mongolia Academy of Agricultural and Animal Husbandry Sciences (protocol code: 2026-0018) and conducted in accordance with the guidelines for the care and use of animals for scientific purposes. Following removal of the head, skin, hooves, and internal organs, the carcass was chilled at 4 °C for 30 min and then weighed. Dressing percentage was calculated according to the following formula:

Dressing percentage (%) = (Carcass weight/Live body weight) × 100%

All lambs were fasted for 24 h and water was withheld for the last 2 h before slaughter. Live body weight was measured immediately prior to fasting. Back-fat thickness (GR) of the carcass was also determined by measuring subcutaneous fat thickness between the 12th and 13th ribs, at a point 11 cm from the dorsal midline, using a vernier caliper.

A sample of the *Longissimus dorsi* muscle (LDM) was separated from each carcass, sub-sampled, and stored in sealed plastic bag at 4 °C for meat quality analysis as described below. Additional sub-samples were collected, frozen in liquid nitrogen, and stored at −80 °C for fatty acid and amino acid analysis.

### 2.4. Analyses

#### 2.4.1. Determination of Serum Antioxidant Activity

The serum samples were analyzed for superoxide dismutase (SOD), malondialdehyde (MDA), glutathione peroxidase (GSH-Px), total antioxidant capacity (T-AOC), and catalase (CAT) using commercial assay kits (Geruisi Biotechnology Co., Ltd., Suzhou, China) according to the manufacturer’s protocols.

#### 2.4.2. Determination of Meat Quality Characteristics

The Longissimus dorsi muscle (LDM) samples were processed and analyzed for the following meat quality traits. Three replicate analyses were conducted for each sample. Water loss rate, drip loss, and cooking loss were determined according to the methods described in the Chinese agricultural standard NY/T 1333-2007 (*Determination of meat quality for livestock and poultry*) [7]. Specifically, water loss rate was measured using the centrifugal method, drip loss by the suspension method, and cooking loss by the heat treatment method.

Water loss rate: Water loss rate was determined according to the centrifugal method described in the Chinese agricultural standard NY/T 1333-2007 (*Determination of meat quality for livestock and poultry*). An LDM subsample was accurately weighed into a centrifuge tube and centrifuged at 1500× *g* for 30 min using an 80-2 electric centrifuge. After centrifugation, excess moisture on the surface of the sample was removed with absorbent paper, and the sample was reweighed. Water loss rate was calculated as:

Water loss rate (%) = [(weight before centrifugation − weight after centrifugation)/weight before centrifugation] × 100%.

Drip Loss: An LDM sample was cut into a block of approximately 5 × 3 × 2.5 cm, weighed, suspended in an aerated polyethylene food bag, and stored at 0–4 °C for 24 h. After storage, the sample was removed, and any surface moisture was gently blotted with absorbent paper before reweighing. Drip loss was calculated as:

Drip loss (%) = [(initial weight − final weight)/initial weight] × 100%

Cooking Loss: Muscle samples were trimmed of tendons and epimysium. A tendons- and epimysium-free LDM sample (120 g) was weighed and placed in a 100 °C water bath for 40 min. The sample was then removed from the water bath, cooled to room temperature (22 °C), and subsequently reweighed. Cooking loss was calculated as follows:

Cooking loss (%) = [(weight before cooking − weight after cooking)/weight before cooking] × 100%

Muscle Shear Force: A subsample of LDM was trimmed of surface fat, sealed in a plastic bag, and stored at 4 °C for 24 h. The bag was then opened, and a thermometer (CEM, Shenzhen Everbest Machinery Industry Co., Ltd., Shenzhen, China) was inserted into the geometric center of the sample. The bag was resealed and placed in an 80 °C water bath for 30 min. After removal from the water bath and cooling to room temperature, the sample was cut into three strips (approximately 3.0 cm × 1.0 cm × 1.0 cm) along the muscle fiber orientation. Each strip was measured for shear force using a C-LM digital muscle tenderness meter (Beijing Tianxiang Feiyu Technology Co., Ltd. (TENOVO), Beijing, China) [8].

Muscle pH: The pH of the LDM was measured using a pH-STAR direct-reading pH meter (Matthäus, Germany) by inserting the electrode probe 2 cm below the sample surface at 45 min post-slaughter (pH_45min_) and after 24 h of storage at 4 °C (pH_24h_).

Muscle Color: The LDM sample was cut into 5 cm × 5 cm blocks and the reflectance color of the meat block was measured using a CR410 colorimeter (Konica Minolta, Tokyo, Japan) according to the method described in NY/T 1333-2007. Measurements are expressed in the Hunter system with a standard white plate as reference. Each sample was measured three times, and the average value was used to determine L*, a*, and b* where L* is lightness (L* = 100, white; L* = 0, dark), and a* and b* are the intensity of the different colors (a* > 0, redness; a* < 0, greenness; b* > 0, yellowness; b* < 0, blueness) [9].

#### 2.4.3. Determination of Muscle Composition

The LDM sample was initially freeze-dried at −100 °C for 48 h, then dried in an air-forced oven at 105 °C to constant weight. Moisture content was calculated as the difference between the initial and final weights after drying. The sample was also analyzed for crude protein according to GB 5009.5-2016 (National Food Safety Standard—Determination of Protein in Foods), crude fat according to GB/T 5009.6-2008 (Determination of Fat in Foods), and ash according to GB 5009.4-2016 (National Food Safety Standard—Determination of Ash in Foods). Total phosphorus content was determined according to the Chinese national standard GB/T 9695.4-2009 (Meat and meat products—Determination of total phosphorus content). Briefly, the LDM sample was digested with a mixture of sulfuric acid and hydrogen peroxide in a digestion block at 250 °C for 2 h and the absorbance density of the digested clear solution was measured at a wavelength of 420 nm using an ultraviolet spectrophotometer (UV-1780, Shimadzu Corporation, Kyoto, Japan). 

Amino acid analysis: Amino acids were analyzed by high-performance liquid chromatography (HPLC) following the national standard method GB 5009.124-2016. Briefly, the LDM sample was hydrolyzed with 6 M HCl at 110 °C for 24 h under nitrogen atmosphere. After derivatization with o-phthalaldehyde (OPA), the amino acids were separated on a C18 column (4.6 × 250 mm, 5 μm) at 40 °C. The mobile phase consisted of 0.1 M sodium acetate (pH 7.2) and methanol (60:40, *v*/*v*) at a flow rate of 1.0 mL/min. Detection was performed at 338 nm using a diode array detector (DAD). The analysis was conducted by LC-Bio Technology Co., Ltd. (Hangzhou, China).

Fatty acid analysis: Fatty acids were analyzed by gas chromatography–mass spectrometry (GC-MS) following the national standard method GB 5009.168-2016. Briefly, lipids were extracted from the LDM sample using a chloroform–methanol (2:1, *v*/*v*) solution, followed by methylation with 14% boron trifluoride–methanol reagent. The fatty acid methyl esters (FAMEs) were separated on a fused-silica capillary column (HP-88, 100 m × 0.25 mm × 0.2 μm). The oven temperature was programmed from 80 °C to 230 °C. Helium was used as the carrier gas at a flow rate of 1.0 mL/min. Detection was performed using a mass spectrometer in electron impact (EI) mode. The analysis was conducted by LC-Bio Technology Co., Ltd. (Hangzhou, China).

### 2.5. Determination of Rumen Bacterial Microbiome

#### 2.5.1. DNA Extraction, Library Preparation, and High-Throughput Sequencing

Rumen bacterial DNA was extracted using a commercial DNA extraction kit (Novogene Co., Ltd., Beijing, China), and the concentration and purity of the extracted bacterial DNA were assessed using a Nanodrop spectrophotometer (Thermo Fisher Scientific, Waltham, MA, USA). The V4 region of the 16S rRNA gene was amplified by PCR with the 515F and 806R primers (Table 3). Amplification products were verified by 2% agarose electrophoresis and then used for library construction of the sequencing library. The libraries were subjected to quality control and quantification using Qubit fluorescence quantification and qPCR. High-throughput sequencing was performed on the Illumina HiSeq 2500 platform (Illumina, Inc., San Diego, CA, USA). All PCR amplifications and sequencing were conducted by Novogene Co., Ltd. (Beijing, China).

#### 2.5.2. Sequencing Date Processing and Bioinformatic Analysis

Raw sequencing data were first demultiplexed and assigned to each sample based on their unique barcodes and primer sequences. Barcode and primer sequences were then removed. Cutadapt (version 4.0) was then used to identify and remove reverse primer sequences (forward primers had already been removed during demultiplexing). The resulting raw tags were then subjected to quality control and filtering using FASTP (version 0.23.1) with the following parameters: minimum mean quality score of Q20, maximum 40% of bases below Q20, and minimum read length of 100 bp after filtering. The high-quality clean tags were then denoised using the DADA2 algorithm to infer amplicon sequence variants (ASVs). Chimeric sequences were detected and removed by alignment against the Silva database (version 138). Finally, taxonomic assignment was performed using the Silva database (version 138) with a confidence threshold of 0.8.

A Venn diagram visually represented the shared and unique distribution of bacterial community compositions across different fermented *Caragana korshinskii* supplementation levels. The Venn diagram was plotted using the VennDiagram package in R, while the flower diagram was generated with the SVG module in Perl.

Traditional OTU clustering (e.g., 97% similarity) was not performed. Alpha diversity of the bacterial community was analyzed on the QIIME2 platform, with specific metrics including observed ASVs, Chao1 index, Shannon index, and Simpson index. Rarefaction curves were further generated using R (version 4.0.3) to evaluate sequencing depth adequacy and species diversity coverage. Alpha diversity indices were compared among groups using the Kruskal–Wallis test, followed by Dunn’s post hoc test with Benjamini–Hochberg false discovery rate correction for multiple comparisons. To assess the effects of different fermented *Caragana korshinskii* supplementation levels on the similarity of rumen bacterial community composition in Mongolian sheep, beta diversity analysis was performed based on the QIIME2 platform. Beta diversity was assessed using PERMANOVA (Adonis) with 999 permutations based on Bray–Curtis distances. Principal Coordinates Analysis (PCoA) was computed and visualized using the ade4 and ggplot2 packages in R (version 4.0.3).

### 2.6. Statistical Analysis

The experimental unit for all analyses was the pen (*n* = 12), with 3 pens per treatment group. For growth performance and serum parameters measured in all 36 lambs, pen means (average of 3 lambs per pen) were calculated prior to analysis. For slaughter performance, meat quality, muscle composition, and fatty acid profiles measured in one lamb per pen, the individual value was used to represent the pen. All data were analyzed using one-way analysis of variance (ANOVA) with pen as the experimental unit. Orthogonal polynomial contrasts were used to test for linear and quadratic effects of increasing dietary levels of fermented *Caragana korshinskii*. Post hoc comparisons among treatment means were performed using Tukey’s honest significant difference (HSD) test. Differences were considered significant at *p* < 0.05. Spearman correlation analysis was conducted to assess the relationships between the relative abundance of rumen bacteria (at the phylum and genus levels) and the concentrations of amino acids and fatty acids in the Longissimus dorsi muscle. Only microbial taxa and metabolites that showed significant differences among treatment groups in the ANOVA were included in the correlation analysis. Prior to analysis, all data were normalized using Z-score transformation. For compositional data (microbial relative abundances), centered log-ratio (CLR) transformation was applied. Multiple testing correction was performed using the false discovery rate (FDR) method (Benjamini–Hochberg procedure). Correlations with an adjusted *p* < 0.05 were considered statistically significant. All statistical analyses were performed using SPSS 23.0 (IBM Corp., Armonk, NY, USA) and R software (version 4.0.3).

## 3. Results

### 3.1. Growth Performance of Lambs Fed Diets Containing Different Levels of Fermented Caragana korshinskii

Lambs in the CK10 had heavier (*p* < 0.05) final body weight (LW) at the end of the 105 d feeding trial and greater (*p* < 0.05) ADG than those in the C group, but similar final LW and ADG to that of CK15 and CK20 lambs (Table 4). No difference was observed in final LW and ADG among lambs fed C, CK15 and CK20. The lambs in CK10 group also ate more (*p* < 0.05) that lambs in C and CK20, but no difference was observed among the C, CK15 and CK20. All lambs had similar FCR.

### 3.2. Effects of Different Inclusion Levels of Fermented Caragana korshinskii on Serum Antioxidant Indices in Mongolian Sheep

As shown in Table 5, there were no significant differences in serum antioxidant parameters among all groups at the beginning of the experiment (*p* > 0.05). At the end of the trial, no significant differences were observed in GSH-Px levels among groups (*p* > 0.05), while T-AOC in CK10, CK15, and CK20 was significantly higher than that in C (*p* < 0.05). CAT activity in CK10 was significantly increased compared with C (*p* < 0.05). Furthermore, SOD activity in CK10 and CK15 was significantly higher than that in both C and CK20 (*p* < 0.05). MDA concentrations in CK10, CK15, and CK20 were significantly lower than those in C (*p* < 0.05).

### 3.3. Effects of Different Dietary Levels of Fermented Caragana korshinskii on Slaughter Performance in Mongolian Sheep

As presented in Table 6, dietary supplementation with varying levels of fermented *Caragana korshinskii* significantly influenced carcass weight in Mongolian sheep (*p* < 0.05). In contrast, no significant differences were detected in dressing percentage, GR value, or backfat thickness among treatment groups (*p* > 0.05). Specifically, carcass weight was significantly greater in CK10 compared with the C (*p* < 0.05).

### 3.4. Effects of Different Dietary Levels of Fermented Caragana korshinskii on Meat Quality in Mongolian Sheep

As shown in Table 7, dietary supplementation with varying levels of fermented *Caragana korshinskii* significantly affected water loss rate and drip loss in the *longissimus dorsi* muscle of Mongolian sheep (*p* < 0.05). In contrast, cooking loss did not differ among groups (*p* > 0.05). The water loss rate was significantly higher in the C and CK20 groups than in the CK10 and CK15 groups (*p* < 0.05). Drip loss was also significantly greater in the C group compared with the CK10 group (*p* < 0.05).

Shear force was similarly influenced by dietary treatment (*p* < 0.05), with the C and CK20 groups exhibiting significantly higher values than the CK10 and CK15 groups (*p* < 0.05). Significant differences in pH were observed 45 min post-slaughter (*p* < 0.05), where the C group had a higher pH than the CK15 group. All values at this time point remained within the normal range for meat color (pH 6.0–7.0). After 24 h, no intergroup differences in pH were detected (*p* > 0.05), with all values staying above 5.0. The ΔpH was greater in the CK10 group than in the CK15 group (*p* < 0.05); the subsequent decline in pH to approximately 5.0 did not adversely affect meat quality and was consistent with normal aging.

Dietary treatments also significantly influenced meat color parameters (L*, a*, b*) (*p* < 0.05). Lightness (L*) was lower in CK10 than in C. Redness (a*) was higher in CK10 compared with C and CK20. Yellowness (b*) was lower in CK10 and CK15 relative to C and CK20 (all *p* < 0.05).

### 3.5. Results of Muscle Nutritional Composition Indicators

As shown in Table 8, dietary supplementation with varying levels of fermented *Caragana korshinskii* had no significant effect (*p* > 0.05) on the moisture, crude fat, crude protein, crude ash, or total phosphorus content of the *longissimus dorsi* muscle in Mongolian sheep.

### 3.6. Effects of Different Dietary Levels of Fermented Caragana korshinskii on Muscle Amino Acid Composition in Mongolian Sheep

As shown in Table 9, the concentrations of non-essential, sweet, bitter, umami, branched-chain, and functional amino acids in the *longissimus dorsi* muscle of Mongolian sheep were not significantly influenced by dietary inclusion of fermented Caragana at different levels (*p* > 0.05).

Within the non-essential amino acids, aspartic acid content was significantly higher in CK15 compared with C (*p* < 0.05) and markedly higher than in CK10 and CK20 (*p* < 0.01). In contrast, glutamic acid content was lower in C relative to all experimental groups (*p* < 0.05).

Among essential amino acids, threonine concentration was elevated in CK15 compared with C and other experimental groups (*p* < 0.05). Isoleucine content was lower in C and CK10 than in CK15 and CK20 (*p* < 0.05). Furthermore, leucine content was significantly lower in C, CK10 and CK15 compared with CK20 (*p* < 0.05).

No significant differences were detected for the remaining amino acids analyzed (*p* > 0.05).

### 3.7. Effects of Different Dietary Levels of Fermented Caragana korshinskii on Muscle Fatty Acid Composition in Mongolian Sheep

As shown in Table 10, the composition of fatty acids varied among the experimental groups. In terms of saturated fatty acids, the content of stearic acid was significantly higher in CK10 compared with C, CK15, and CK20 (*p* < 0.05).

Regarding unsaturated fatty acids, the linoleic acid content in CK10 was significantly elevated relative to all other groups. The difference reached statistical significance when compared with CK20 (*p* < 0.05) and was highly significant in comparison with C and CK15 (*p* < 0.01). Similarly, the α-linolenic acid content was higher in CK10 than in C, CK15, and CK20. This increase was significant compared to C (*p* < 0.05) and highly significant compared to both CK15 and CK20 (*p* < 0.01).

No significant differences were detected in the remaining fatty acids across the groups (*p* > 0.05).

### 3.8. Effects of Different Fermented Caragana korshinskii Levels on the Rumen Microbial Community in Mongolian Sheep

#### 3.8.1. Analysis of Venn Diagram Results of Rumen Microbiota in Mongolian Sheep Fed Different Dietary Levels of Fermented *Caragana korshinskii* (*n* = 12 Pens)

**Figure 1:**
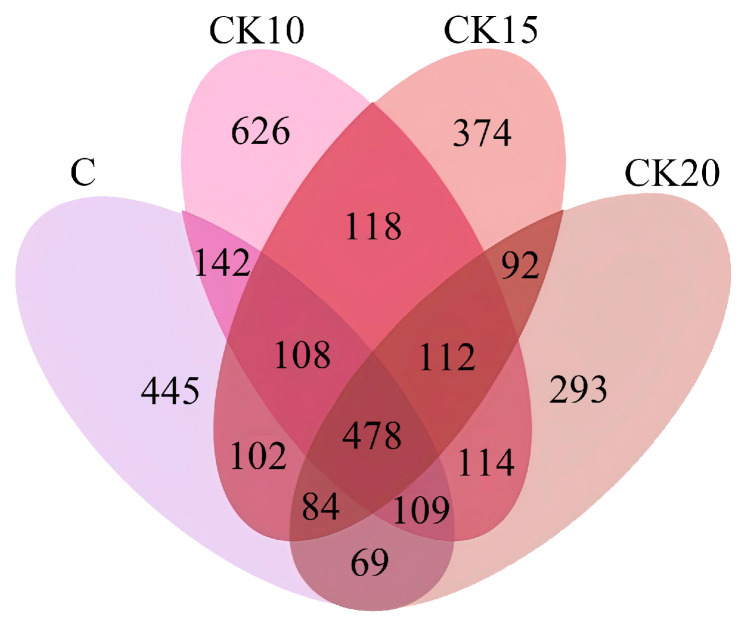
Venn diagram of rumen microorganisms. Each color represents a different treatment group: C (purple), CK10 (pink), CK15 (red), and CK20 (brown). Overlapping regions indicate shared ASVs between groups.

To examine the effects of graded levels of fermented *Caragana korshinskii* on the rumen microbial community, rumen fluid samples were collected from 12 sheep and analyzed via 16S rDNA sequencing. Following quality filtering and denoising, 3293 amplicon sequence variants (ASVs) were identified. The ASV distribution among the experimental groups was as follows: C (839 ASVs), CK10 (801 ASVs), CK15 (554 ASVs), and CK20 (909 ASVs). A core set of 478 ASVs was common to all four groups (Figure 1).

#### 3.8.2. Analysis of Alpha Diversity of Rumen Microbiota in Mongolian Sheep Fed Different Dietary Levels of Fermented *Caragana korshinskii* (*n* = 12 Pens)

**Figure 2:**
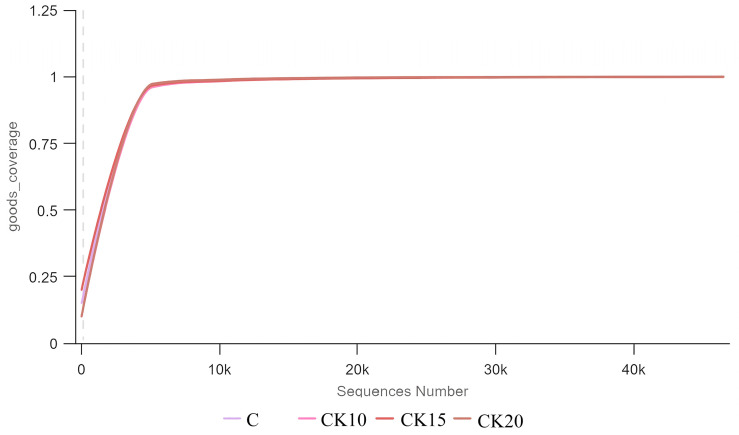
Shannon diversity rarefaction curve.

The sample-based rarefaction curve analysis demonstrated that as sequencing depth (x-axis) increased, the Shannon diversity index approached a plateau and the number of observed ASVs exhibited a progressively decelerating increase before stabilizing (Figure 2). This pattern indicates that the sequencing depth was adequate to capture the majority of microbial taxa present in the samples, with the observed data approaching saturation. These results confirm that the sequencing data robustly represent the in situ bacterial community structure and support their suitability for subsequent microbial diversity analyses in this study.

**Figure 3:**
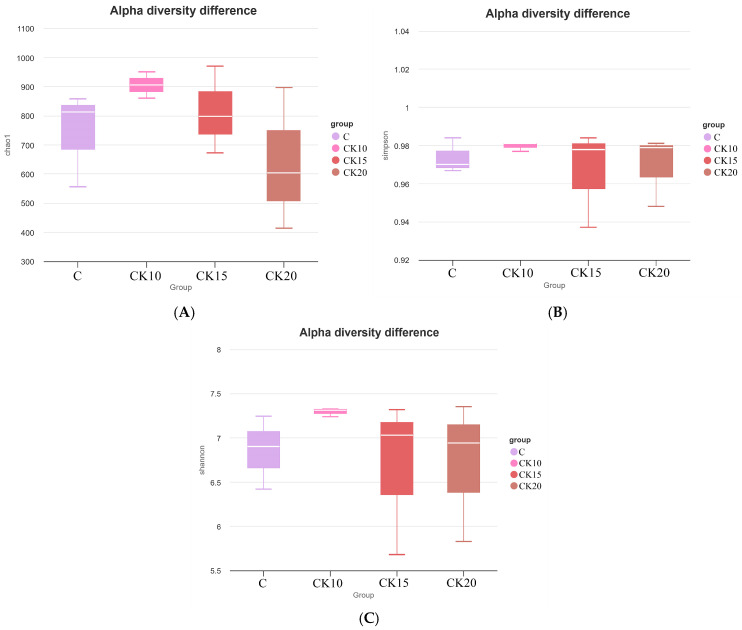
(**A**) Chao1. (**B**) Shannon. (**C**) Simpson.

Alpha diversity of the rumen microbiota was evaluated using the Chao1, Shannon, and Simpson indices to characterize microbial richness and diversity. As shown in Figure 3, no significant differences (*p* > 0.05) were observed among the dietary groups supplemented with varying levels of fermented *Caragana korshinskii*. Although statistical significance was not reached, the Chao1 index indicated a numerically higher microbial richness in the EG1 group compared to other groups (Figure 3A).

#### 3.8.3. Analysis of Beta Diversity of Rumen Microbiota in Mongolian Sheep Fed Different Dietary Levels of Fermented *Caragana korshinskii* (*n* = 12 Pens)

**Figure 4:**
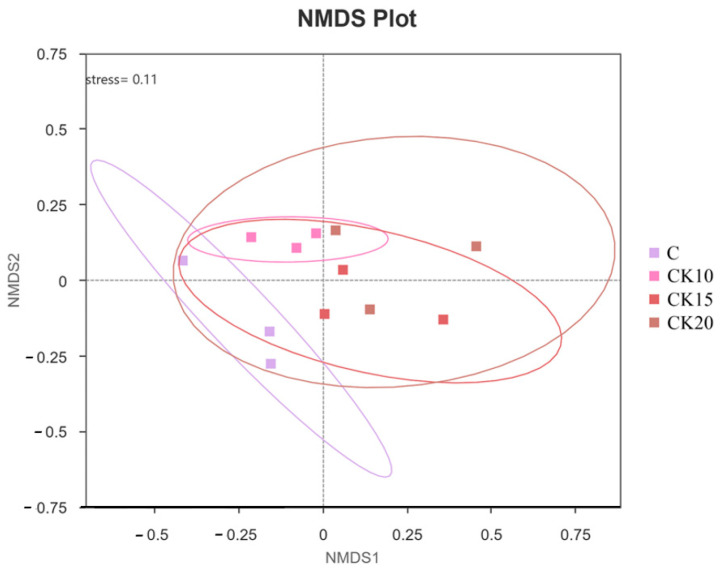
NMDS plot based on Bray–Curtis distance.

Non-metric multidimensional scaling (NMDS) based on Bray–Curtis dissimilarity revealed no distinct clustering among the C, CK10, CK15, and CK20 groups (Figure 4), indicating substantial overlap in overall rumen microbial community composition. Consistent with this pattern, the Kruskal–Wallis test showed no statistically significant differences (*p* > 0.05) in alpha diversity indices—including the Chao1 (richness), Shannon, and Simpson (diversity) indices—across dietary treatments with varying levels of fermented *Caragana korshinskii* supplementation. These results collectively demonstrate that dietary inclusion of fermented *Caragana korshinskii*, at the administered levels, did not significantly alter the richness or diversity of the rumen microbiota (*p* > 0.05).

#### 3.8.4. Composition and Structure of Rumen Microbiota at the Phylum Level in Mongolian Sheep Fed Different Dietary Levels of Fermented *Caragana korshinskii* (*n* = 12 Pens)

As shown in Table 11, the relative abundance of Thermodesulfobacteriota was significantly higher in CK10 and CK20 compared with C and CK15 (*p* < 0.05). Furthermore, the relative abundance of Methanobacteriota in CK10 was significantly higher than that in all other groups. The difference reached an extremely significant level compared with C and CK15 (*p* < 0.01), and a significant level compared with CK20 (*p* < 0.05). No significant differences were detected in the relative abundances of the remaining phyla among the groups (*p* > 0.05).

#### 3.8.5. Composition and Structure of Rumen Microbiota at the Genus Level in Mongolian Sheep Fed Different Dietary Levels of Fermented *Caragana korshinskii*

As shown in Table 12, significant differences were observed in the relative abundances of several microbial genera among the experimental groups. The relative abundance of *Xylanibacter* was significantly higher in CK20 compared to C, CK10, and CK15. Specifically, the difference was significant versus CK15 (*p* < 0.05) and extremely significant versus C and CK10 (*p* < 0.01). Conversely, the relative abundance of *Rikenellaceae_RC9_gut_group* was significantly lower in CK20 than in C, CK10, and CK15 (*p* < 0.05). For *Methanobrevibacter*, CK10 exhibited a significantly higher relative abundance compared to C, CK15, and CK20, with an extremely significant difference versus C and CK15 (*p* < 0.01) and a significant difference versus CK20 (*p* < 0.05). Regarding *Succiniclasticum*, the relative abundances in C and CK10 were significantly lower than those in CK15 and CK20 (*p* < 0.05). No significant differences were detected in the relative abundances of the remaining genera (*p* > 0.05).

### 3.9. Association Analysis Between Rumen Microbial Community and Meat Quality Nutritional Composition

#### 3.9.1. Association Between Rumen Microbiota at the Phylum Level and Muscle Fatty Acid/Amino Acid Composition (*n* = 12 Pens)

**Figure 5:**
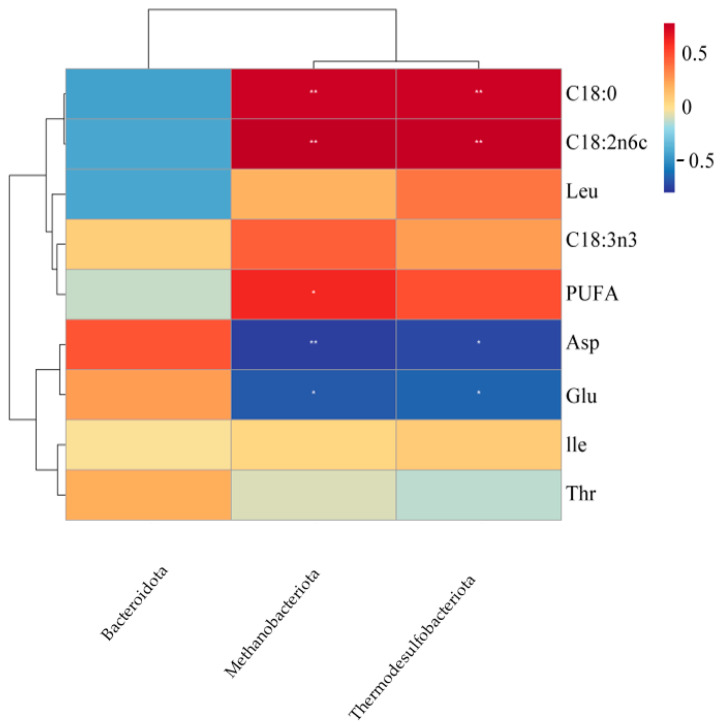
Relationships between rumen microbiota at the phylum level and muscle fatty acid and amino acid composition. * *p* < 0.05; ** *p* < 0.01.

At the phylum level, the association between the rumen microbiota and the composition of muscle fatty acids and amino acids was analyzed, with the results presented in Figure 5. Thermodesulfobacteriota showed significant negative correlations with both glutamic acid and aspartic acid (*p* < 0.05), and highly significant positive correlations with stearic acid (C18:0) and linoleic acid (C18:2n6c) (*p* < 0.01). Methanobacteriota exhibited significant negative correlations with glutamic acid (*p* < 0.05) and aspartic acid (*p* < 0.01). Additionally, it was significantly positively correlated with PUFA (*p* < 0.05), and highly significantly positively correlated with stearic acid (C18:0) and linoleic acid (C18:2n6c) (*p* < 0.01).

#### 3.9.2. Association Between Rumen Microbiota at the Genus Level and Muscle Fatty Acid/Amino Acid Composition (*n* = 12 Pens)

**Figure 6:**
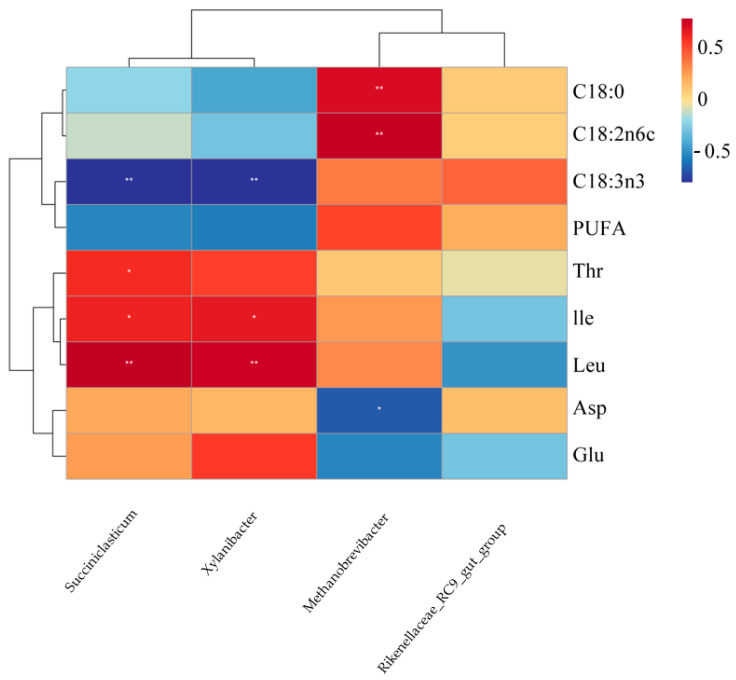
Relationships between rumen microbiota at the genus level and muscle fatty acid and amino acid composition. * *p* < 0.05; ** *p* < 0.01.

The association between the rumen microbiota at the genus level and the composition of muscle fatty acids and amino acids was analyzed, with the results presented in Figure 6. *Xylanibacter* and *Succiniclasticum* exhibited significant positive correlations with both leucine (Leu) and isoleucine (Ile). The correlation with leucine was highly significant (*p* < 0.01), whereas that with isoleucine was significant (*p* < 0.05). In contrast, *Xylanibacter* showed an extremely significant negative correlation with α-linolenic acid (C18:3n3) (*p* < 0.01). Similarly, *Succiniclasticum* was also highly significantly negatively correlated with α-linolenic acid (*p* < 0.01). *Methanobrevibacter* was significantly negatively correlated with aspartic acid (Asp) (*p* < 0.05) and highly significantly positively correlated with both stearic acid (C18:0) and linoleic acid (C18:2n6c) (*p* < 0.01).

#### 3.9.3. Correlation Analysis Between Meat Quality and Muscle Fatty Acid and Amino Acid Composition (*n* = 12 Pens)

**Figure 7:**
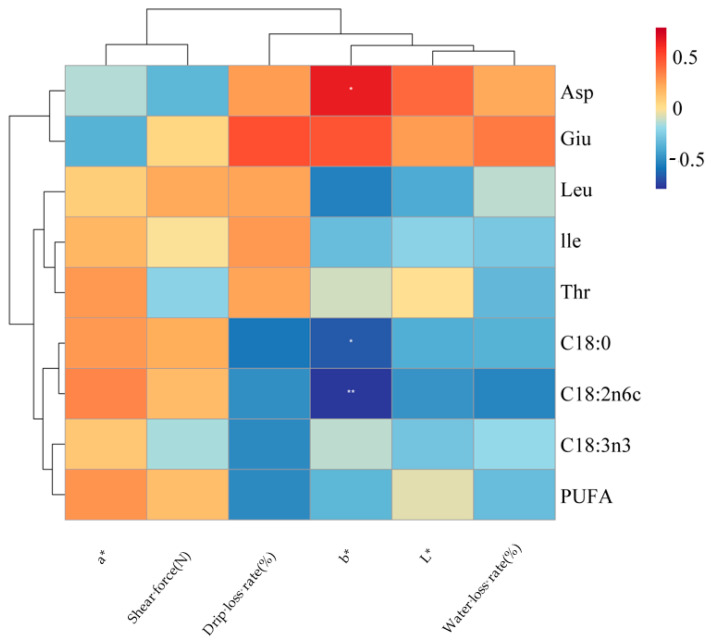
Correlation Analysis between Meat Quality and Muscle Fatty Acid and Amino Acid Composition. * *p* < 0.05; ** *p* < 0.01.

An association analysis was conducted between meat quality and the composition of muscle fatty acids and amino acids, with the results presented in Figure 7. Drip loss showed a significant negative correlation with stearic acid (C18:0) (*p* < 0.05). The yellowness (b*) value was significantly positively correlated with aspartic acid (Asp) and significantly negatively correlated with both stearic acid (C18:0) and linoleic acid (C18:2n6c) (*p* < 0.05), with the correlation with linoleic acid reaching a highly significant level (*p* < 0.01).

## 4. Discussion

The present study demonstrated that dietary inclusion of 10% fermented *Caragana korshinskii* significantly enhanced the final body weight, ADG, and ADFI of Mongolian sheep. This finding is consistent with previous studies showing that solid-state fermentation effectively degrades lignocellulose, enriches microbial protein, and enhances the palatability of straw-based forages, thereby improving nutrient availability and animal growth performance [10,11,12]. Our results extend this paradigm by quantifying the specific growth response in Mongolian sheep and, more critically, by identifying a precise optimal inclusion level for this fermented feed resource. The growth promotion observed at the 10% inclusion level can be mechanistically attributed to a synergistic interplay of factors. First, the fermentation process improved feed palatability and texture, which directly stimulated voluntary intake. Second, the condensed tannins in *Caragana korshinskii* likely exerted a beneficial rumen-bypass protein-protective effect at this moderate level, optimizing the supply of amino acids for post-ruminal absorption and potentially improving nitrogen utilization efficiency [13,14]. Notably, our ruminal microbiota analysis provides direct evidence supporting enhanced fermentation efficiency. The significant increases in the relative abundances of *Firmicutes* (a phylum encompassing many fibrolytic and energy-harvesting bacteria), *Succinivibrionaceae* (implicated in succinate production), and *Prevotella* (versatile degraders of non-cellulosic polysaccharides) collectively indicate a shift in the microbial community towards taxa proficient in energy metabolism and carbohydrate fermentation [15]. This shift aligns with and biologically explains the improved growth performance, offering a deeper insight beyond mere intake metrics. A key contribution of this study is the identification of a clear dose-dependent response, which refines the application guidelines for fermented *Caragana korshinskii*. Contrary to a linear positive relationship, increasing the fermented *Caragana korshinskii* inclusion level to 15% and 20% significantly depressed both ADG and ADFI, establishing 10% as the optimal level under our experimental conditions. This reversal highlights that the benefits of fermentation are constrained by the underlying chemical composition of the substrate when included at high proportions. The decline in performance at supra-optimal levels can be attributed to two predominant limiting factors. First, physical and chemical constraints related to fiber became more pronounced: the elevated intake of neutral detergent fiber (NDF) and acid detergent fiber (ADF) likely enhanced physical fill, limiting dry matter intake [16], while the associated lignin barrier further impeded the digestibility of the fibrous matrix. Second, the cumulative effects of anti-nutritional factors, primarily condensed tannins, transitioned from beneficial to detrimental. At high concentrations, excessive tannins form stable complexes not only with dietary proteins but also with endogenous digestive enzymes, thereby impairing nutrient digestibility and metabolic utilization [17]. This mechanistic explanation is corroborated by the distinct shifts in specific ruminal microbial taxa observed in the 20% fermented *Caragana korshinskii* group, suggesting a potential inhibitory effect of tannin overload on key rumen microbiome functions. In summary, the impact of fermented *Caragana korshinskii* on Mongolian sheep operates within a narrow optimal window (approximately 10% of the diet). Within this window, it enhances growth by synergistically improving palatability, providing rumen-protected protein, and fostering a rumen microbial ecosystem geared towards efficient energy harvest. However, beyond this threshold, the benefits are overridden by fiber overload and tannin toxicity. This well-defined dose–response relationship, elucidated through integrated analysis of performance, nutrient composition, and rumen microbiota, is a significant contribution. It moves beyond merely confirming the value of fermentation and provides crucial, precise scientific guidance for the safe and effective utilization of *Caragana korshinskii* and similar tannin-rich, unconventional feed resources in ruminant production systems. Mechanistically, the moderate concentration of condensed tannins in the 10% fermented *Caragana korshinskii* group may have formed reversible complexes with dietary proteins, protecting them from ruminal degradation and increasing the supply of amino acids for post-ruminal absorption. This ‘rumen-bypass protein’ effect, together with the flavonoid-mediated modulation of rumen microbiota (e.g., increased *Methanobrevibacter* abundance), likely contributed to the improved growth performance observed at this inclusion level.

This study systematically investigated the dose-dependent effects of dietary supplementation with fermented *Caragana korshinskii* on antioxidant function in Mongolian sheep. By evaluating T-AOC, key antioxidant enzyme activities, and lipid peroxidation biomarkers, we demonstrated that fermented *Caragana korshinskii* significantly enhanced systemic antioxidant status in a dose-dependent manner. Specifically, the 10% supplementation group exhibited a comprehensive improvement, with serum T-AOC, SOD, GSH-Px, and CAT activities being significantly higher than those in the control group. This coordinated upregulation indicates an enhanced capacity to scavenge ROS. Correspondingly, serum MDA content—a key marker of lipid peroxidation—was significantly reduced across all supplementation groups, with the most pronounced decrease observed at the 10% inclusion level. These results collectively confirm that moderate fermented *Caragana korshinskii* supplementation effectively alleviates systemic oxidative stress in Mongolian sheep. The observed enhancement in antioxidant capacity can be primarily attributed to the phenolic compounds, particularly tannins, present in *Caragana korshinskii*, which are well-established antioxidants [18,19,20]. Beyond directly neutralizing free radicals, these bioactive compounds are known to modulate endogenous antioxidant defense systems, potentially through the activation of signaling pathways such as Nrf2/ARE, thereby promoting the synthesis and activity of enzymes including SOD and GSH-Px [21]. The significant increase in T-AOC in our study supports this mechanism of systemic antioxidant activation. Our findings align with previous reports on the antioxidant properties of plant polyphenols in ruminants but extend this knowledge by delineating a clear dose–response relationship under practical feeding conditions. Notably, consistent with the growth performance trends reported in the companion section of this study, the antioxidant benefits diminished at higher inclusion levels. In the 15% fermented *Caragana korshinskii* group, only T-AOC and SOD remained elevated compared to the control, whereas in the 20% fermented *Caragana korshinskii* group, only T-AOC was significantly higher. This attenuation at elevated doses may be explained by two interrelated factors. First, high dietary fiber levels can induce digestive and metabolic stress, potentially increasing endogenous ROS production and thereby counteracting the benefits of phenolic antioxidants [22]. Second, excessive tannins may interfere with the absorption or utilization of essential micronutrients that act synergistically in antioxidant defense, such as selenium and vitamin E [23]. Our results thus highlight a nonlinear response, where beyond an optimal threshold, the potential pro-oxidant or antinutritional effects of high-fiber, high-tannin feeds may emerge. To our knowledge, this is the first study to establish a clear dose–response relationship between fermented *Caragana korshinskii* supplementation and antioxidant function in Mongolian sheep. While previous research has documented the antioxidant potential of polyphenol-rich forage, our work systematically quantifies the optimal inclusion level (10% in this model) that maximizes antioxidant benefits without inducing negative effects. Furthermore, we provide mechanistic insight by linking the observed phenotypic improvements to both direct free radical scavenging and the probable activation of endogenous defense pathways, while also explaining the decline in efficacy at higher doses through digestive and nutritional antagonism. In summary, 10% fermented *Caragana korshinskii* supplementation most effectively enhanced the antioxidant function of Mongolian sheep, likely through a combination of direct radical neutralization and upregulation of endogenous enzyme systems. The diminished benefits at higher inclusion levels underscore that excessive fiber and condensed tannins can themselves become sources of metabolic stress. Therefore, precise dosage control is critical to harnessing the full antioxidant value of this fermented feed resource in ruminant nutrition. These findings offer practical guidance for developing antioxidant-rich feeding strategies to improve oxidative status and overall health in grazing livestock.

This study demonstrated that dietary inclusion of fermented *Caragana korshinskii* had only limited effects on carcass performance in Mongolian sheep. A significant increase in carcass weight was observed only at the 10% inclusion level, whereas key carcass indicators—including dressing percentage and meat yield—remained unchanged across all treatment groups. These findings suggest that fermented *Caragana korshinskii* influenced nutrient deposition rates during the growth phase without systematically altering final body composition or slaughter outcomes [24]. The elevated carcass weight in the 10% fermented *Caragana korshinskii* group corresponds directly to the higher average daily gain recorded during the finishing period, indicating a transient improvement in growth performance [25]. This effect may be attributed to enhanced nutrient utilization efficiency mediated by phenolic compounds released through fermentation, which likely supported greater protein deposition at this specific inclusion level [26]. The absence of significant differences in most carcass metrics may be explained by several factors. First, traits such as dressing percentage are largely determined by genetic background and pre-slaughter body condition, rendering them less responsive to moderate dietary fiber variation compared to dynamic growth parameters [27]. This observation aligns with earlier reports in other sheep breeds, where *Caragana korshinskii* supplementation similarly failed to alter dressing percentage [28,29]. Second, even after fermentation, the inherently lignified structure of *Caragana korshinskii* may retain a ruminal filling effect, potentially restricting voluntary intake of higher-energy feed components. This may have limited the divergence in final body condition and carcass grading among groups within the experimental timeframe [30]. While previous studies have examined raw or fermented shrub forage in ruminant nutrition, the present investigation specifically evaluates fermented *Caragana korshinskii* in the context of Mongolian sheep—a breed adapted to sparse grazing resources. Our results extend existing knowledge by demonstrating that fermented *Caragana korshinskii* can enhance finishing-phase growth efficiency without substantially altering carcass composition. This supports its potential as a strategic feed ingredient in regions where *Caragana korshinskii* is widely available. Within the 10–20% dietary inclusion range, the principal benefit of fermented *Caragana korshinskii* appears to be the promotion of growth rate during finishing, rather than the modulation of carcass traits. For producers, this suggests that fermented *Caragana korshinskii* may improve feeding efficiency without compromising carcass yield. Future research should investigate whether extended finishing periods or optimized combinations with energy-dense supplements can enhance the carcass quality benefits of fermented *Caragana korshinskii*, particularly in terms of meat fatty acid profile or sensory attributes.

Meat quality is a critical determinant of both the economic value and consumer acceptance of mutton [31,32]. The present study investigated the effects of dietary supplementation with fermented *Caragana korshinskii* on the meat quality characteristics of Mongolian sheep. Our findings reveal distinct, dose-dependent improvements in key physical attributes, advancing the understanding of how fermented forage resources can be utilized to enhance ovine meat quality. Notably, fermented *Caragana korshinskii* supplementation significantly improved meat color and tenderness. The most pronounced color enhancement was observed in the 10% inclusion group, which exhibited a higher a* value alongside lower L* and b* values, indicative of a more stable and desirable red meat color. This improvement is likely attributable to an enhanced systemic antioxidant capacity, as supported by previous studies linking dietary antioxidants to myoglobin stability and reduced metmyoglobin formation [21,33]. Concurrently, shear force was significantly reduced, and water-holding capacity (WHC) was improved in both the 10% and 15% fermented *Caragana korshinskii* groups, reflecting enhanced tenderness. The observed reduction in shear force may be associated with enhanced proteolytic activity during the aging process, leading to improved myofibrillar fragmentation [34]. Furthermore, the slower rate of early post-mortem pH decline in the 15% fermented *Caragana korshinskii* group suggests a modulation of glycolytic metabolism, which can mitigate protein denaturation and preserve ultrastructural integrity, thereby improving WHC [35,36,37]. In contrast to the marked physical improvements, the proximate composition of the *Longissimus dorsi* muscle—including moisture, crude protein, and crude fat content—remained unaffected by fermented *Caragana korshinskii* inclusion. This result aligns with earlier ruminant studies reporting that forage-based interventions often alter meat physical properties without changing its proximate composition [38]. It underscores that the primary mechanism of action for fermented *Caragana korshinskii* is likely structural and physicochemical rather than nutritive, focusing on post-mortem muscle biochemistry and protein functionality. The differential response patterns among quality indicators underscore the mechanistic complexity of dietary interventions. While tenderness improved at both 10% and 15% inclusion levels, optimal color stabilization was specific to the 10% dose. This divergence suggests that the physiological pathways governing myoglobin oxidation and those regulating proteolysis and glycolytic flux possess distinct sensitivities to the bioactive compounds present in fermented *Caragana korshinskii*. Such a dose-specific effect highlights the importance of precision in dietary formulation to target specific meat quality outcomes. In conclusion, this study demonstrates that dietary inclusion of 10% fermented *Caragana korshinskii* effectively enhances the eating quality of Mongolian sheep meat, primarily through improved color stability and tenderness. These improvements can directly increase market appeal and consumer satisfaction. Our work contributes to the field by elucidating how a novel fermented feed resource can be strategically applied to optimize meat physical attributes without altering its fundamental nutritional profile, offering a practical approach to adding value in sheep production systems. Future research should focus on elucidating the specific bioactive compounds in fermented *Caragana korshinskii* responsible for these effects and validating the findings under diverse production conditions.

Muscle amino acid composition is a critical determinant of both the nutritional quality and sensory attributes of mutton [39]. In this study, we systematically quantified 16 amino acids in the *Longissimus dorsi* of Mongolian sheep fed diets supplemented with fermented *Caragana korshinskii*. While the overall amino acid profile—including total essential amino acids (EAAs), flavor-associated amino acids, and branched-chain amino acids (BCAAs)—remained unchanged, fermented *Caragana korshinskii* supplementation induced targeted, dose-dependent alterations in specific amino acids. This finding indicates that, within the 10–20% inclusion range, FCK does not disturb systemic protein metabolism, which is primarily governed by genetic and physiological homeostasis. The stability in broad amino acid categories contrasts with the specific modulations observed in key individual amino acids, highlighting a refinement in muscle amino acid “quality” rather than a shift in total “quantity.” First, within the non-essential amino acid pool, aspartic acid was significantly elevated at the 15% inclusion level, while glutamic acid increased across all fermented *Caragana korshinskii*-supplemented groups. Both amino acids serve as crucial intermediates in the TCA cycle and as nitrogen donors for amino acid biosynthesis [40]. Their enrichment suggests that fermented *Caragana korshinskii* may enhance precursor availability for muscle metabolism, possibly through improved ruminal nitrogen utilization or enhanced hepatic transamination capacity. Second, a graded regulation of essential amino acid deposition was evident. Threonine increased specifically at the 15% inclusion level, whereas isoleucine and leucine were significantly elevated only at the 20% level [41,42]. This stepwise response suggests that different inclusion rates differentially influence the post-ruminal supply of EAAs, likely by modulating microbial protein synthesis or intestinal absorption patterns [43]. Our results align with previous studies reporting that certain feed additives can selectively alter muscle amino acid profiles [40], but extend this knowledge by demonstrating a clear dose-dependent specificity for threonine and BCAAs in sheep muscle. A key contribution of this work lies in demonstrating that fermented *Caragana korshinskii* can selectively enrich functional and flavor-related amino acids—such as aspartic acid, glutamic acid, threonine, and key BCAAs—without altering the overall protein nutritional value. This targeted enrichment offers a potential strategy to enhance the flavor profile and functional properties of mutton, while maintaining its fundamental nutritional composition. Future studies should focus on elucidating the precise mechanisms, including rumen microbial dynamics and post-absorptive metabolism, underlying these observed amino acid responses.

The fatty acid composition of muscle tissue is a critical determinant of both the nutritional value and sensory attributes of meat [44]. Our findings demonstrate that dietary supplementation with fermented *Caragana korshinskii* significantly altered the muscle fatty acid profile in Mongolian sheep. Notably, the 10% inclusion level of fermented *Caragana korshinskii* elicited a distinct regulatory effect, characterized by a selective modification of saturated fatty acid (SFA) deposition alongside the preservation and enhancement of beneficial unsaturated fatty acids. A key outcome was the specific elevation in stearic acid (C18:0) content observed in the 10% fermented *Caragana korshinskii* group. This elevation in stearic acid, a long-chain SFA, suggests a diet-induced shift in lipid metabolism [45]. While elevated stearic acid is often associated with increased dietary SFA intake, our data indicate a more complex metabolic adjustment. We hypothesize that FCK may modulate key hepatic metabolic pathways, such as de novo lipogenesis and stearoyl-CoA desaturase (SCD) activity, which regulate the conversion of stearic acid to oleic acid (C18:1 n-9) [46]. Importantly, this shift did not compromise the growth performance advantages concurrently observed in this group, differentiating our results from studies where negative growth-performance trade-offs accompanied similar FA profile changes. Supplementation with 10% fermented *Caragana korshinskii* supplementation significantly increased the muscle content of linoleic acid (C18:2 n-6) and α-linolenic acid (C18:3 n-3)—essential polyunsaturated fatty acids (PUFAs) for human health. This dual enhancement is particularly significant, as it suggests that fermented *Caragana korshinskii* may partially protect dietary PUFAs from ruminal biohydrogenation [47], a process that typically limits their deposition in ruminant meat. Furthermore, we propose that the previously documented antioxidant properties of fermented feed ingredients likely contributed to enhanced PUFA retention in muscle tissue by mitigating post-absorptive lipid peroxidation. The co-elevation of stearic acid and essential PUFAs in the 10% fermented *Caragana korshinskii* group points to a multi-targeted regulatory effect on lipid metabolism, influencing ruminal biohydrogenation, intestinal absorption, and post-absorptive synthesis and oxidation pathways. This coordinated response indicates that at its optimal inclusion level for growth, fermented *Caragana korshinskii* can synergistically improve growth performance and enhance the health-promoting fatty acid profile of the meat—an outcome not always achieved simultaneously in animal nutrition interventions [48]. In conclusion, the dietary incorporation of 10% fermented *Caragana korshinskii* effectively reshapes the muscle fatty acid composition in Mongolian sheep. The primary nutritional advancement lies in the significant enrichment of meat with essential PUFAs. The concurrent rise in stearic acid—which exhibits a neutral cholesterolemic effect in humans and serves as a direct precursor for endogenous oleic acid synthesis—represents a metabolically balanced outcome rather than a detrimental increase in SFAs. This study thus provides a novel nutritional strategy for producing functional mutton enriched with beneficial fatty acids, leveraging an unconventional fermented feed resource to achieve dual benefits in both animal performance and product quality.

The rumen microbial ecosystem is fundamental to the efficient degradation of fibrous plant materials in ruminants [49]. Our study employed 16S rDNA sequencing to delineate how dietary incorporation of fermented *Caragana korshinskii* modulates the rumen microbiota of Mongolian sheep. A key finding was the absence of significant changes in alpha diversity indices across treatment groups. This indicates that fermented *Caragana korshinskii*, within the 10–20% inclusion range, did not induce a wholesale restructuring of the microbial community. Instead, it elicited a shift in the relative abundances of specific bacterial and archaeal populations, suggesting a targeted mechanism of “functional enrichment”—a nuanced shift in the functional capacity of the microbiota without altering its overall structural complexity. Consistent with the established literature, the rumen bacterial community was dominated by the phyla *Bacteroidota* and *Bacillota* across all diets [50,51,52], affirming the stability of the core microbial architecture. Significant shifts, however, were observed among non-dominant taxa, underscoring the specific effect of the diet. Notably, the 10% FCK inclusion significantly elevated the relative abundance of the methanogenic archaeal phylum *Methanobacteriota*. This precise modulation highlights how dietary intervention can selectively influence key functional guilds, such as methanogens, even amidst overall community stability. At the genus level, fermented *Caragana korshinskii* demonstrated clear dose-dependent regulation of functionally important taxa. In the 10% fermented *Caragana korshinskii* group, the increased abundance of *Methanobrevibacter*—a primary hydrogenotrophic methanogen [53,54]—aligned with the rise in *Methanobacteriota*. This co-enrichment suggests an optimized ruminal hydrogen economy and a tight coupling between acidogenesis and methanogenesis. This metabolic efficiency likely contributed to the highest growth performance observed at this inclusion level, despite the potential energy diversion into methane. Higher inclusion levels (15% and 20%) promoted a distinct functional shift, enriching taxa associated with fiber degradation and shifts in fermentation pathways. The specific enrichment of *Xylanibacter* in the 20% group represents a direct microbial response to the increased xylan content supplied by fermented *Caragana korshinskii* [55]. Furthermore, the significant rise in *Succiniclasticum* abundance in the 15% and 20% groups implies a metabolic shift toward propionate-type fermentation [56]. While this shift could enhance the supply of glucogenic precursors, our data suggest that its potential benefits were likely counterbalanced by the physical limitations and reduced digestibility associated with high-fiber diets, as reflected in the observed growth performance outcomes. Broader adjustments in the rumen microenvironment in response to dietary fiber modification were indicated by changes in other taxa. The increase in *Thermodesulfobacteriota* (10% and 20% groups) and the decrease in *Rikenellaceae*_RC9_gut_group (20% group) may reflect alterations in redox potential and nutrient flow [57], further illustrating the rumen’s adaptive response. In conclusion, this study elucidates that fermented *Caragana korshinskii* primarily modulates the functional landscape, rather than the structural diversity, of the rumen microbiota. The 10% inclusion level optimized overall fermentation homeostasis and hydrogen metabolism, which underpinned the peak growth performance. At higher inclusions, despite the enrichment of specialized fiber-degrading and propionate-producing taxa, the metabolic advantages were offset by the inherent constraints of high dietary fiber. These findings provide a “novel microbial mechanistic explanation” for the previously identified growth performance “optimal window.” They go beyond simply describing compositional changes to proposing a concept of functional enrichment, thereby offering a refined rationale for improving the utilization of *Caragana korshinskii* through strategic rumen microbial management. The dose-dependent response—beneficial at 10% but diminished at 15% and 20%—can be explained by the dual role of tannins. At low to moderate concentrations, tannins exert beneficial effects by modulating rumen fermentation and protecting dietary proteins. However, at higher concentrations, they may bind to endogenous digestive enzymes and dietary nutrients, reducing nutrient digestibility. This biphasic response is characteristic of many plant-derived bioactive compounds.

The rumen microbiota functions as the central metabolic interface in ruminants, serving as the critical link between dietary digestion and host nutrient deposition [58]. This study specifically examined how shifts in the rumen microbial community correlate with amino acid and fatty acid profiles in skeletal muscle, thereby providing insights into the microbial regulation of nutrient flow from the rumen to muscle tissue. At the phylum level, both *Thermodesulfobacteriota* and *Methanobacteriota* exhibited significant negative correlations with muscle glutamic acid and aspartic acid content. As hydrogen-utilizing taxa involved in methanogenesis [59] and sulfate reduction [60], their co-enrichment may redirect metabolic hydrogen and carbon fluxes away from pathways supporting microbial protein synthesis, ultimately reducing the supply of these amino acid precursors to peripheral tissues [61]. This suggests that modulating these hydrogen-consuming pathways may represent a strategy for improving nitrogen utilization efficiency in ruminants. In contrast, *Thermodesulfobacteriota* was positively correlated with muscle stearic acid (C18:0) and linoleic acid (C18:2n6). This phylum’s role in maintaining a low redox potential likely promotes ruminal biohydrogenation, facilitating the conversion of unsaturated fatty acids to saturated forms [60]. Similarly, *Methanobacteriota* showed strong positive correlations with both stearic and linoleic acids, which can be attributed to its hydrogenotrophic activity lowering ruminal H_2_ partial pressure, thereby providing a thermodynamic drive for biohydrogenation [62,63,64]. Collectively, these findings indicate that while these phyla may compete for substrates in nitrogen metabolism, they act synergistically in promoting lipid saturation pathways. At the genus level, we identified several key functional associations. *Xylanibacter* (a xylan-degrader) and *Succiniclasticum* (a succinate-to-propionate converter) were positively correlated with muscle leucine and isoleucine content. Their synergistic activities likely optimize carbon and energy availability in the rumen, supporting microbial synthesis of branched-chain amino acids and their subsequent deposition in muscle [65,66,67]. Conversely, both genera were negatively correlated with a-linolenic acid (C18:3n3). We propose that their metabolic functions—fiber degradation and enhanced propionate production—may alter hydrogen flow and redox conditions, thereby accelerating the biohydrogenation of this polyunsaturated fatty acid (PUFA) and reducing its retention in muscle [68,69]. *Methanobrevibacter* mirrored the patterns observed at the phylum level, showing a negative correlation with aspartic acid and strong positive correlations with stearic and linoleic acids. This further corroborates the role of methanogens in driving fatty acid hydrogenation while competing for precursors in amino acid synthesis [70]. In summary, specific rumen microbial taxa demonstrate distinct and sometimes opposing correlations with muscle nutrient profiles. Hydrogen-consuming phyla (*Methanobacteriota*, *Thermodesulfobacteriota*) appear to promote fatty acid biohydrogenation while limiting the availability of precursors for certain amino acids. In contrast, fiber-degrading and propionate-producing genera (*Xylanibacter*, *Succiniclasticum*) support branched-chain amino acid deposition but may reduce PUFA retention in muscle. Compared with earlier studies focusing primarily on ruminal fermentation parameters or microbial community structure, our work directly links microbial taxa and metabolic guilds to muscle nutrient composition, offering a more integrated understanding of the rumen-to-muscle nutrient axis. These findings provide a microbial–ecological basis for explaining how dietary interventions such as fermented *Caragana korshinskii* can influence meat quality, highlighting potential microbial targets for nutritional modulation aimed at optimizing nutrient partitioning in ruminant production. Correlation analysis between meat quality attributes and muscle fatty acids and amino acids composition revealed that drip loss was significantly negatively correlated with stearic acid. This may be attributed to the fact that stearic acid, as a saturated fatty acid, possesses a higher melting point and greater chemical stability, which helps maintain cell membrane integrity, reduce post-slaughter exudative fluid loss, and thus improve water-holding capacity [71]. The b* value was significantly positively correlated with aspartic acid. As a key intermediate in energy metabolism, an increase in aspartic acid content may reflect a more active metabolic state in the muscle, which helps to sustain metmyoglobin reductase activity and thereby stabilize meat color [72]. The b* value was significantly negatively correlated with both stearic acid and linoleic acid, with the correlation with linoleic acid reaching a highly significant level. As a major polyunsaturated fatty acid, linoleic acid is highly susceptible to lipid peroxidation, and its oxidation products can accelerate myoglobin oxidation, leading to meat browning (decreased b* value). The negative correlation between the b* value and stearic acid may indirectly reflect the overall consumption of unsaturated fatty acids through ruminal biohydrogenation.

## 5. Conclusions

This study demonstrated that dietary inclusion of 10% fermented *Caragana korshinskii* optimally enhanced growth performance, antioxidant capacity, and meat quality in Mongolian sheep without compromising carcass traits. Notably, this inclusion level selectively enriched beneficial fatty acids (C18:0, C18:2n6c, and C18:3n3) and functional amino acids (aspartic acid, glutamic acid, leucine, isoleucine) in muscle tissue. Integrated correlation analyses revealed that these nutritional improvements were mechanistically linked to shifts in rumen microbial ecology: hydrogen-consuming phyla (*Methanobacteriota* and *Thermodesulfobacteriota*) correlated with fatty acid saturation, while fiber-degrading genera (*Xylanibacter*, *Succiniclasticum*) associated with branched-chain amino acid deposition. These findings establish fermented *Caragana korshinskii* as a strategic feed resource for producing functional ruminant meat through targeted modulation of the rumen–muscle axis.

## Figures and Tables

**Table 1:** Nutritional Levels of Roughage Ingredients in the Experiment (% DM basis).

Nutrient Levels	*Leymus chinensis* Hay	Fermented *Caragana korshinskii*
Dry matter (DM)	93.88 ± 0.23	45.74 ± 0.12
Crude protein (CP)	9.87 ± 0.61	10.41 ± 0.84
Ether extract (EE)	3.42 ± 0.47	5.13 ± 0.42
Neutral detergent fiber (NDF)	61.62 ± 1.65	69.97 ± 1.47
Acid detergent fiber (ADF)	38.33 ± 1.64	39.68 ± 1.90
Calcium (Ca)	0.27 ± 0.16	2.13 ± 0.09
Phosphorus (P)	0.03 ± 0.02	0.12 ± 0.01
Water-soluble carbohydrates (WSC)		0.23 ± 0.06
PH		3.9
Ammonia nitrogen (NH_3_-N, mg/g)		0.03 ± 0.00
Acetic acid		1.29 ± 0.18
Propionic acid		ND
Isobutyric acid		ND
Butyric acid		ND
Lactic acid		4.92 ± 0.25
True protein		6.43 ± 0.31
Total phenolics (TP)		9.43 ± 0.30
Hydrolyzable tannins (HT)		5.11 ± 0.15
Condensed tannins (CT)		0.48 ± 0.02

**Table 2:** Composition and nutrient levels of experimental diets (% DM basis).

Items	C	CK10	CK15	CK20
Corn grain	25.00	25.00	25.00	25.00
Cottonseed meal	4.00	4.00	4.00	4.00
Wheat bran	10.00	10.00	10.00	10.00
Soybean meal	5.50	5.50	5.50	5.50
Cane molasses	1.50	1.50	1.50	1.50
DDGS	10.00	10.00	10.00	10.00
Limestone	1.00	1.00	1.00	1.00
CaHPO_4_	1.00	1.00	1.00	1.00
NaCl	1.00	1.00	1.00	1.00
Fermented *Caragana korshinskii*	-	10.00	15.00	20.00
*Leymus chinensis* hay	40.00	30.00	25.00	20.00
Premix ^1^	1.00	1.00	1.00	1.00
Total	100.00	100.00	100.00	100.00
Nutrientlevels	-	-	-	-
ME (MJ/kg)	9.75	9.65	9.60	9.55
CP	14.36	14.50	14.58	14.65
Ca	0.49	0.56	0.60	0.63
P	0.44	0.48	0.50	0.52

^1^ ME is a calculated value, computed according to the formula provided in “Nutritional Requirements of Meat Sheep in China,” while other nutritional levels are measured values.

**Table 3:** Primers and sequences.

Classification	Region	Primer Name	Primer Sequence
Bacterium16SrDNA	V4	515F	GTGCCAGCMGCCGCGGTAA
Bacterium16SrDNA	V4	806R	GGACTACHVGGTWTCTAAT

**Table 4:** Growth performance of Mongolian sheep fed diets containing different levels of fermented *Caragana korshinskii* (*n* = 12 pens).

Items	C ^1^	CK10	CK15	CK20	SEM	*p*-Value		
						ANOVA	Linear	Quadratic
Initial body weight (kg)	20.16	20.19	20.42	20.29	0.121	0893	0.595	0.757
Final body weight (kg)	33.73 ^b^	36.83 ^a^	35.55 ^ab^	35.33 ^ab^	0.364	0.005	0.114	0.003
Average daily gain (g/d)	129.30 ^b^	158.44 ^a^	145.56 ^ab^	143.17 ^ab^	3.658	0.024	0.273	0.016
Average Daily Feed Intake (g/d)	1052.81 ^b^	1166.94 ^a^	1101.54 ^ab^	1055.94 ^b^	14.782	0.004	0.534	0.002
Ratio of feed intake to gain	7.99	7.21	7.49	7.19	0.139	0.118	0.071	0.323

^1^ C, Control, lambs fed basal *Leymus chinensis* hay-concentrate diet; CK10, lambs fed diet with fermented *Caragana korshinskii* replacing 10% of *Leymus chinensis* hay in basal diet; CK15, lambs fed diet with fermented *Caragana korshinskii* replacing 15% of *Leymus chinensis* hay in basal diet; CK20, lambs fed diet with fermented *Caragana korshinskii* replacing 20% of *Leymus chinensis* hay in basal diet. ^a,b^ Means in the same row without same superscript letter differ (*p* < 0.05).

**Table 5:** Effects of Different Dietary Levels of Fermented Caragana on Serum Antioxidant Indices in Mongolian Sheep (*n* = 12 pens).

Items	Day	C	CK10	CK15	CK20	SEM	*p*-Value		
							ANOVA	Linear	Quadratic
T-AOC/(U/mL)	0	0.58	0.64	0.65	0.62	0.021	0.727	0.548	0.360
	105	0.56 ^b^	0.80 ^a^	0.79 ^a^	0.72 ^a^	0.029	0.000	0.000	0.000
CAT/(U/mL)	0	1.91	1.84	2.03	1.99	0.052	0.681	0.437	0.902
	105	2.18 ^b^	2.93 ^a^	2.66 ^ab^	2.60 ^ab^	0.108	0.090	0.246	0.051
GSH-Px/(U/mL)	0	81.58	83.07	83.15	79.22	1.414	0.792	0.629	0.410
	105	76.86	132.99	92.58	107.17	12.773	0.512	0.677	0.450
SOD/(U/mL)	0	63.29	65.4	66.37	62.33	1.638	0.854	0.910	0.425
	105	62.44 ^b^	69.74 ^a^	70.24 ^a^	61.15 ^b^	1.440	0.009	0.667	0.001
MDA/(nmol/L)	0	3.32	3.35	3.18	3.15	0.092	0.858	0.466	0.884
	105	4.64 ^a^	3.48 ^b^	3.29 ^b^	3.28 ^b^	0.184	0.000	0.000	0.003

^a,b^ Means in the same row without the same superscript letter differ significantly (*p* < 0.05).

**Table 6:** Effects of Different Dietary Levels of Fermented Caragana on the Slaughter Performance of Mongolian Sheep (*n* = 12 pens).

Items	C	CK10	CK15	CK20	SEM	*p*-Value		
						ANOVA	Linear	Quadratic
Carcass weight (kg)	16.93 ^b^	18.38 ^a^	17.20 ^ab^	17.70 ^ab^	0.191	0.021	0.411	0.131
Dressing percentage (%)	48.98	49.91	48.32	49.01	0.394	0.616	0.694	0.888
Greyhound rib (mm)	18.91	18.32	20.11	21.36	0.538	0.191	0.064	0.361
Thickness of backfat (mm)	5.53	6.20	6.85	6.99	0.401	0.615	0.219	0.761

^a,b^ Means in the same row without the same superscript letter differ significantly (*p* < 0.05).

**Table 7:** Effects of Different Dietary Levels of Fermented Caragana on Meat Quality in Mongolian Sheep (*n* = 12 pens).

Items	C	CK10	CK15	CK20	SEM	*p*-Value		
						ANOVA	Linear	Quadratic
Water loss rate (%)	7.21 ^a^	2.6 ^b^	3.46 ^b^	6.03 ^a^	0.572	0.000	0.045	0.000
Drip loss rate (%)	10.64 ^a^	8.63 ^b^	10.48 ^ab^	10.90 ^ab^	0.313	0.007	0.141	0.009
Cooked meat percentage (%)	45.18	43.44	43.85	44.09	0.391	0.476	0.435	0.239
pH_45min_	6.98	6.78	6.58	6.87	0.059	0.087	0.246	0.034
pH_24h_	5.27	5.33	5.25	5.32	0.022	0.450	0.699	0.901
ΔpH	1.71	1.44	1.32	1.55	0.061	0.122	0.211	0.042
L*	31.41 ^a^	28.76 ^b^	30.49 ^ab^	29.80 ^ab^	0.424	0.122	0.342	0.193
a*	12.47 ^c^	14.33 ^a^	13.72 ^ab^	12.99 ^bc^	0.238	0.005	0.452	0.001
b*	2.52 ^a^	1.70 ^c^	2.11 ^b^	1.75 ^c^	0.109	0.001	0.002	0.044
Shear force (N)	65.03 ^a^	61.32 ^b^	62.24 ^b^	65.12 ^a^	0.424	0.000	0.045	0.000

^a–c^ Means in the same row without the same superscript letter differ significantly (*p* < 0.05).

**Table 8:** Effects of Different Dietary Levels of Fermented Caragana on the Nutrient Content of Muscle in Mongolian Sheep (%, *n* = 12 pens).

Items	C	CK10	CK15	CK20	SEM	*p*-Value		
						ANOVA	Linear	Quadratic
Moisture	74.93	74.39	74.97	74.84	0.217	0.831	0.892	0.690
EE	1.65	2.10	1.66	1.55	0.123	0.423	0.509	0.276
CP	16.63	15.91	16.79	16.44	0.134	0.081	0.752	0.409
Ash	1.99	2.22	2.05	1.95	0.065	0.619	0.678	0.305
TP	0.18	0.16	0.17	0.182	0.005	0.404	0.813	0.139

**Table 9:** Effects of Different Dietary Levels of Fermented Caragana on Muscle Amino Acid Composition in Mongolian Sheep (µmol/kg, *n* = 12 pens).

Items	C	CK10	CK15	CK20	SEM	*p*-Value		
						ANOVA	Linear	Quadratic
NEAA	-	-	-	-	-			
Asp	6.37 ^b^	2.98 ^c^	9.10 ^a^	4.06 ^c^	0.973	0.000	0.667	0.014
Ser	12.59	14.67	16.87	16.6	1.179	0.609	0.235	0.649
Clu	6.59 ^b^	9.47 ^a^	8.77 ^a^	8.47 ^a^	0.352	0.002	0.230	0.123
Pro	0.96	0.70	1.07	1.64	0.169	0.297	0.142	0.237
Ala	28.02	29.80	38.23	36.73	1.941	0.155	0.049	0.634
Tyr	10.72	12.7	11.91	13.3	0.843	0.780	0.423	0.877
Arg	6.27	5.8	7.55	6.46	0.500	0.717	0.648	0.785
Gly	93.37	45.23	95.63	100.07	14.024	0.531	0.600	0.389
EAA	-	-	-	-				
Thr	7.04 ^c^	8.49 ^bc^	21.35 ^a^	10.82 ^b^	1.722	0.000	0.000	0.000
Val	13.93	14.77	14.67	16.37	0.654	0.667	0.281	0.764
Ile	11.90 ^b^	12.90 ^b^	14.50 ^a^	14.41 ^a^	0.338	0.000	0.000	0.046
Leu	24.01 ^b^	24.59 ^b^	25.15 ^b^	30.55 ^a^	0.810	0.000	0.000	0.000
Phe	14.33	18.47	14.53	17.57	0.866	0.226	0.435	0.735
Met	5.06	6.26	5.51	6.99	0.401	0.383	0.191	0.863
Lys	6.63	7.86	8.17	8.2	0.493	0.704	0.323	0.587
Trp	4.96	5.64	5.1	5.55	0.208	0.672	0.560	0.815
TAA	254.13	228.13	297.09	302.11	17.145	0.405	0.197	0.659
DAA	12.87	11.85	20.88	17.78	1.661	0.172	0.103	0.728
SAA	150.48	111.42	179.47	176.19	16.544	0.494	0.363	0.609
BAA	85.83	99.23	91.64	102.59	3.732	0.429	0.235	0.873
BCAA	49.43	56.00	52.13	58.27	1.843	0.719	0.818	0.434
FAA	140.51	92.68	162.29	161.04	16.048	0.426	0.385	0.486

Note: Umami amino acids (DAA) were calculated as the sum of aspartic acid and glutamic acid; sweet-tasting amino acids (SAA) as glycine, alanine, serine, proline, lysine and threonine; bitter-tasting amino acids (BAA) as histidine, arginine, tyrosine, valine, methionine, isoleucine, leucine and phenylalanine; branched-chain amino acids (BCAA) as valine, isoleucine and leucine; functional amino acids (FAA) as aspartic acid, glycine, glutamic acid, alanine and arginine; and total amino acids (TAA) as the sum of non-essential amino acids (NEAA) and essential amino acids (EAA). ^a–c^ Means in the same row without the same superscript letter differ significantly (*p* < 0.05).

**Table 10:** Effects of Different Dietary Levels of Fermented Caragana on Muscle Fatty Acid Composition in Mongolian Sheep (µg/g, *n* = 12 pens).

Items	C	CK10	CK15	CK20	SEM	*p*-Value		
						ANOVA	Linear	Quadratic
SFA	2723.57	4209.99	1934.38	2819.66	358.182	0.142	0.486	0.087
C6:0	0.34	0.28	0.30	0.24	0.021	0.589	0.250	0.989
C8:0	1.99	3.30	1.29	1.01	0.404	0.169	0.140	0.282
C10:0	20.10	38.40	13.15	12.29	4.938	0.215	0.256	0.313
C11:0	0.15	0.33	0.13	0.20	0.041	0.404	0.899	0.539
C12:0	11.30	23.25	8.07	12.66	2.937	0.317	0.669	0.529
C13:0	0.47	1.05	0.35	0.66	0.150	0.412	0.932	0.668
C14:0	220.00	368.67	142.40	256.00	41.843	0.311	0.748	0.831
C15:0	13.40	21.72	9.83	12.70	2.542	0.436	0.555	0.606
C16:0	1780.00	2373.00	1189.00	1616.67	0.400	0.444	0.863	0.142
C17:0	35.83	53.80	24.50	25.97	6.156	0.341	0.299	0.401
C18:0	634.50 ^bc^	1320.64 ^a^	542.21 ^c^	876.50 ^b^	96.803	0.000	0.886	0.056
C20:0	3.25	3.61	1.50	2.39	0.504	0.495	0.329	0.799
C21:0	0.35	0.37	0.30	0.45	0.037	0.600	0.496	0.421
C22:0	0.49	0.61	0.27	0.58	0.078	0.492	0.930	0.581
C23:0	0.37	0.38	0.31	0.47	0.034	0.487	0.448	0.336
C24:0	1.08	1.12	0.76	0.88	0.067	0.197	0.114	0.782
MUFA	2846.42	3572.36	2016.88	2533.51	336.944	0.479	0.437	0.400
C14:1	10.03	18.05	5.48	5.93	2.367	0.436	0.555	0.606
C16:1	241.00	324.00	165.00	200.67	31.543	0.353	0.335	0.708
C17:1	4.45	4.80	2.77	2.58	0.544	0.383	0.144	0.804
C18:1n9t	21.93	29.50	13.39	14.20	3.478	0.352	0.228	0.630
C18:1n9c	2563.33	3190.00	1826.67	2306.67	300.089	0.499	0.458	0.908
C20:1	5.68	6.01	3.58	3.47	0.643	0.390	0.147	0.865
PUFA	731.61 ^b^	853.74 ^a^	661.38 ^c^	709.48 ^bc^	22.967	0.001	0.019	0.001
C18:2n6t	1.57	1.93	1.30	1.11	0.160	0.317	0.176	0.387
C18:2n6c	368.00 ^c^	507.76 ^a^	362.50 ^c^	425.44 ^b^	17.656	0.000	0.260	0.000
C18:3n6	8.83	10.16	7.96	6.36	0.734	0.343	0.164	0.327
C18:3n3	33.20 ^b^	44.05 ^a^	23.70 ^c^	24.35 ^c^	2.578	0.000	0.000	0.014
C20:2	3.49	3.20	2.46	2.25	0.245	0.243	0.058	0.932
C20:3n6	18.9	20.3	16.67	13.01	1.424	0.315	0.116	0.376
C20:4n6	274.33	248.00	230.00	222.33	8.089	0.077	0.015	0.482
C20:5n3	13.83	12.00	10.52	8.54	0.930	0.238	0.052	0.966
C22:6n3	9.45	7.11	6.26	6.53	0.714	0.416	0.165	0.381

^a–c^ Means in the same row without the same superscript letter differ significantly (*p* < 0.05).

**Table 11:** Effects of different dietary levels of fermented Caragana on the rumen microbiota at the phylum level in Mongolian sheep (% top6, *n* = 12 pens).

Items	C	CK10	CK15	CK20	SEM	*p*-Value		
						ANOVA	Linear	Quadratic
Bacteroidota	57.72	53.79	61.92	51.28	0.031	0.763	0.746	0.664
Bacillota	31.80	32.42	26.71	34.08	0.022	0.778	0.963	0.537
Synergistota	0.97	1.42	3.94	4.03	0.011	0.259	0.493	0.211
Thermodesulfobacteriota	0.83 ^b^	1.56 ^a^	0.88 ^b^	1.44 ^a^	0.000	0.001	0.024	0.391
Methanobacteriota	1.87 ^c^	7.23 ^a^	2.09 ^c^	4.80 ^b^	1.018	0.001	0.201	0.054
Verrucomicrobiota	0.56	0.48	1.37	2.20	0.000	0.275	0.080	0.494
Others	1.20	1.85	0.65	2.52	0.000	0.355	0.419	0.423

Note: Data are sorted in descending order of abundance. The same sorting method applies to the table below. ^a–c^ Means in the same row without the same superscript letter differ significantly (*p* < 0.05).

**Table 12:** Effects of different dietary levels of fermented Caragana on the rumen microbiota at the genus level in Mongolian sheep (% top17, *n* = 12 pens).

Items	C	CK10	CK15	CK20	SEM	*p*-Value		
						ANOVA	Linear	Quadratic
*Xylanibacter*	12.93 ^c^	7.16 ^c^	22.49 ^b^	33.83 ^a^	0.031	0.000	0.000	0.001
*Quinella*	15.26	8.31	8.99	19.60	0.033	0.413	0.572	0.129
*Rikenellaceae*_*RC9*_*gut*_*group*	10.22 ^a^	11.93 ^a^	11.17 ^a^	2.59 ^b^	0.009	0.000	0.000	0.000
*Others*	10.37	11.46	6.32	7.09	0.012	0.725	0.663	0.990
*unclassified*_*Muribaculaceae*	4.49	12.85	5.18	7.46	0.011	0.124	0.910	0.232
*unclassified*_*F082*	5.96	10.34	3.48	5.27	0.013	0.241	0.395	0.576
*Methanobrevibacter*	0.48 ^c^	7.21 ^a^	2.08 ^c^	4.79 ^b^	0.012	0.000	0.013	0.006
*Prevotellaceae*_*UCG-001*	2.71	1.39	5.52	2.94	0.008	0.725	0.663	0.990
*Prevotellaceae*_*UCG-003*	4.49	2.40	2.10	1.29	0.012	0.266	0.075	0.566
*Fretibacterium*	0.93	1.38	3.69	3.98	0.013	0.639	0.244	0.967
*Succiniclasticum*	1.55 ^b^	1.95 ^b^	3.66 ^a^	4.28 ^a^	0.000	0.005	0.001	0.802
*Desulfovibrio*	4.83	1.80	0.65	0.93	0.007	0.502	0.192	0.493
*Christensenellaceae*_*R-7*_*group*	1.76	1.87	1.01	1.28	0.002	0.409	0.220	0.838
unclassified_*WCHB1-41*	0.56	0.47	1.35	2.17	0.001	0.269	0.078	0.494
*unclassified*_*Bacteroidales*_*RF16_group*	1.26	1.51	1.49	0.26	0.001	0.306	0.214	0.396
*NK4A214*_*group*	1.24	1.74	0.85	0.63	0.000	0.049	0.034	0.175
*Prevotellaceae*_*NK3B31_group*	0.65	1.81	0.53	1.04	0.000	0.334	0.966	0.531

^a–c^ Means in the same row without the same superscript letter differ significantly (*p* < 0.05).

## Data Availability

The data that support the findings of this study are available from the corresponding author upon reasonable request.

## Abbreviations

The following abbreviations are used in this manuscript:

FLC	Forage–Livestock Conflict
DM	Dry matter
NDF	Neutral detergent fiber
ADF	Acid detergent fiber
T-AOC	total antioxidant capacity
SOD	Serum superoxide dismutase
MDA	malondialdehyde
GSH-Px	glutathione peroxidase
CAT	catalase
TMR	total mixed rations
ADG	Average daily gain
ADMI	Average daily dry matter intake
FCR	Feed conversion ratio
GR	Greyhound rib
DAA	Umami amino acids
SAA	sweet-tasting amino acids
BAA	bitter-tasting amino acids
BCAA	branched-chain amino acids
FAA	functional amino acids
TAA	total amino acids
NEAA	non-essential amino acids
EAA	essential amino acids
